# Heterogeneity of Glia in the Retina and Optic Nerve of Birds and Mammals

**DOI:** 10.1371/journal.pone.0010774

**Published:** 2010-06-17

**Authors:** Andy J. Fischer, Christopher Zelinka, Melissa A. Scott

**Affiliations:** Department of Neuroscience, College of Medicine, The Ohio State University, Columbus, Ohio, United States of America; University of Oldenburg, Germany

## Abstract

We have recently described a novel type of glial cell that is scattered across the inner layers of the avian retina [1]. These cells are stimulated by insulin-like growth factor 1 (IGF1) to proliferate, migrate distally into the retina, and up-regulate the nestin-related intermediate filament transitin. These changes in glial activity correspond with increased susceptibility of neurons to excitotoxic damage. This novel cell-type has been termed the Non-astrocytic Inner Retinal Glia-like (NIRG) cells. The purpose of the study was to investigate whether the retinas of non-avian species contain cells that resemble NIRG cells. We assayed for NIRG cells by probing for the expression of Sox2, Sox9, Nkx2.2, vimentin and nestin. NIRG cells were distinguished from astrocytes by a lack of expression for Glial Fibrilliary Acidic Protein (GFAP). We examined the retinas of adult mice, guinea pigs, dogs and monkeys (*Macaca fasicularis*). In the mouse retina and optic nerve head, we identified numerous astrocytes that expressed GFAP, S100β, Sox2 and Sox9; however, we found no evidence for NIRG-like cells that were positive for Nkx2.2, nestin, and negative for GFAP. In the guinea pig retina, we did not find astrocytes or NIRG cells in the retina, whereas we identified astrocytes in the optic nerve. In the eyes of dogs and monkeys, we found astrocytes and NIRG-like cells scattered across inner layers of the retina and within the optic nerve. We conclude that NIRG-like cells are present in the retinas of canines and non-human primates, whereas the retinas of mice and guinea pigs do not contain NIRG cells.

## Introduction

The retinas of vertebrates contain many different types of glial cells. The primary activities of these glia include retinal homeostasis and support of neuronal function. Across all vertebrate species, retinal glia include Müller glia, derived from retinal stem cells, and microglia, derived from hematopoietic stem cells. With some variations between species, retinal glia can include astrocytes and oligodendrocytes. For example, the avascular retinas of chickens, guinea pigs and rabbits contain oligodendrocytes that myelinate the axons of ganglion cells in the nerve fiber layer (NFL) [2], [3], [4]. Although vascular retinas contain significant numbers of astrocytes that are closely associated with the blood vessels [5], [6], [7], avascular retinas contain few, if any, astrocytes [1], [8].

In the chicken eye, we have recently identified a novel type of glial cell that is scattered across inner retinal layer [1]. These cells were termed Non-astrocytic Inner Retinal Glial (NIRG) cells. The NIRG cells express vimentin, Sox2 and Sox9, similar to Müller glia and retinal progenitors, but these cells do not express other glial markers such as Top_AP_, glutamine synthetase or high levels of glial fibrilliary acidic protein (GFAP). We found that IGF1 stimulated the NIRG cells to proliferate, migrate distally into the retina, and up-regulate transitin, an intermediate filament orthologous to mammalian nestin. Further, IGF1 stimulated microglia to acquire a reactive morphology and up-regulate CD45 and lysosomal membrane glycoprotein. The IGF1 receptor was expressed only by presumptive NIRG cells and microglia that were scattered across inner retinal layers. With glial cells stimulated by IGF1, there were elevated levels of cell death and widespread focal retinal detachments in response to an excitotoxic insult. The increased cell death was prominent within areas of retinal detachment which were coincident with a stark loss of Müller glia and an accumulation of NIRG cells. Taken together, these findings indicate that NIRG cells are a novel type of retinal cell that is sensitive to IGF1 and whose activity impacts the survival of retinal neurons and Müller glia. At the time of hatching, NIRG cells are scattered across all regions of the retina, with greater abundance in central regions, and this distribution remains unchanged during the first 4 weeks of postnatal development [1].

There have been no studies that examine whether NIRG cells are present in the retinas of non-avian vertebrates. Accordingly, in this study we test the hypothesis that NIRG-like cells are present in the retinas of mice, guinea pigs, dogs and non-human primates. In addition, we probe for NIRG-like cells in the optic nerve and nerve head of chickens, mice, guinea pigs, dogs and macaque monkeys.

## Results

For all results described herein, observations were made on tissues obtained from mature animals with normal, healthy retinas. Therefore, descriptions of the different types of glial cells represent the phenotypes of stable, mature, non-reactive cells. In the retinas of species examined in these studies, astrocytes were identified based on expression of GFAP and other markers known to be expressed by mature retinal glia including Sox2, Sox9 and S100β [1], [9], [10], [11], [12], [13], [14]. We probed for NIRG cells immunolabeling for Sox2, Sox9, Nkx2.2, vimentin and transitin/nestin, and an absence of GFAP [1]. Although the homeodomain transcription factors Sox2 and Sox9, and the intermediate filament transitin/nestin are best-known to be expressed by neural and glial progenitors, many recent studies have indicated that the expression of these genes persists in mature, post-mitotic, functional glial cells in the retina of birds and mammals [1], [9], [10], [11], [12], [13], [14]. Nkx2.2, a homeodomain transcription factor, that is best-known to be expressed by oligodendrocyte precursors in the developing spinal cord [15], [16], [17], and by oligodendrocyte precursors in the developing chick visual system [18].

### Glial cells in the optic nerve of chickens

Our previous work has demonstrated that NIRG cells are scattered across central and peripheral regions of the retina; these cells are predominantly found in the distal third of the IPL and in the GCL [1]. We have observed that NIRG cells migrate into the retina from the optic nerve (Zelinka, Scott and Fischer, unpublished), similar to the astrocytes and oligodendrocytes that are found in the avian retina [18], [19]. However, the distribution of NIRG cells in the optic nerve of post-hatch chickens has not been reported. Accordingly, we assayed for NIRG cells and oligodendrocytes in the optic nerve and optic nerve head (ONH) of the post-hatch chicken.

**Figure 1:**
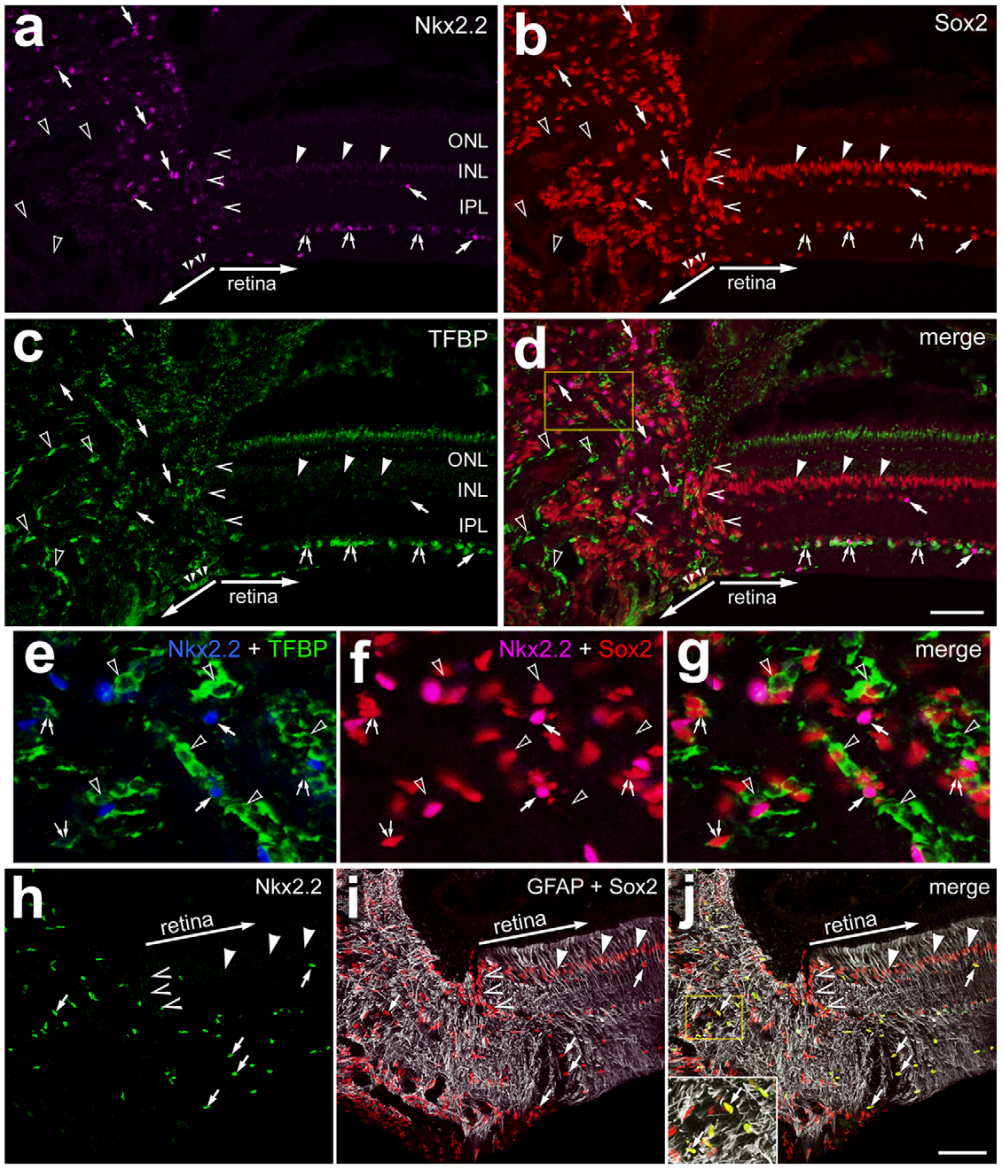
Glial cells in the chick optic nerve are immunoreactive for Nkx2.2, Sox2, Sox9, GFAP and TFBP. Longitudinal sections through the optic nerve and nerve head were labeled with antibodies to Nkx2.2 (magenta in **a**, **d**, **f** and **g**; blue in **e;** green in **h** and **j**), Sox2 (red), TFBP (green in **c**–**e** and **g**) and GFAP (grayscale; **i** and **j**). Images were obtained by using wide-field epifluoresence microscopy (**a–g**) or confocal microscopy (**h–j**). The region indicated by the yellow box in panel **d** is enlarged 4-fold in panels **e–g**, and the region in panel **j** is enlarged 2-fold in the inset. Arrow-heads indicate the nuclei of GFAP/Sox2-positive Müller glia. Small double-arrows indicate TFBP-positive oligodendrocytes in the retina. Arrows indicate Sox2/Nkx2.2-positive nuclei of NIRG cells in the optic nerve and retina. Carets indicate Sox2-positive nuclei of peripapillary glia. Hollow arrow-heads indicate Sox2/Nkx2.2-negative, TFBP-positive oligodendrocytes in the optic nerve. Small double-arrows indicate TFBP-positive oligodendrocytes in the retina. The scale bar (50 µm) in panel d applies to **a–d**, and the bar in **j** applies to **h–j**. Abbreviations: ONL – outer nuclear layer, INL – inner nuclear layer, IPL – inner plexiform layer.

We found numerous NIRG cells within the chick optic nerve and nerve head (Fig. 1). The NIRG cells were identified by co-expression of Nkx2.2 and Sox2, and an absence of GFAP and absence of transferrin binding protein (TFBP). Oligodendrocytes in the avian central nervous system can be identified by immunolabeling for TFBP [20], [21], [22]. Although a few of the TFBP^+^ oligodendrocytes within the GCL were Nkx2.2-positive (Figs. 1a-d), none (n = 312) of the TFBP^+^ oligodendrocytes in the optic nerve were immunoreactive for Nkx2.2 (Figs. 1a-g). Consistent with the pattern of expression in the retina, a small minority (8.2±1.9%) of the TFBP^+^ cells in the optic nerve were immunoreactive for Sox2; the majority of the Sox2^+^ cells in the optic nerve were negative for (or expressed very low levels of) TFBP (Figs. 1e-g). Immunoreactivity for GFAP was elevated in Müller glia that were located within 200 µm of the ONH (Figs. 1e-g). Within the optic nerve and nerve head numerous cells were intensely immunoreactive for GFAP, and most of these cells expressed Sox2 (Figs. 1h-j) and Pax2 (not shown). However, there were numerous Sox2^+^ cells in the optic nerve that were negative for Nkx2.2 (Figs. 1h-j). The Sox2^+^/GFAP^+^/Nkx2.2^−^ cells were likely to be optic nerve astrocytes. In addition, we identified numerous cells in the optic nerve and nerve head that were positive for Sox2/Nkx2.2 and negative for GFAP (Figs. 1h-j); these cells were likely to be NIRG cells. See table 1 for a summary of markers expressed by glial cells in the chick eye, and table 2 for a summary of the types of glial cells in the retina, optic nerve and nerve head.

**Table 1:** Summary of the glial cell types in the eyes of different vertebrate species.

Cell type	Chicken	Mouse	Guinea Pig	Dog	Monkey
Müller glia	numerous	numerous	numerous	numerous	numerous
Retinal astrocytes	rare	many	none	many	many
Retinal oligodendrocytes	some	none	some	none	none
Retinal NIRG cells	many	none	none	many	many
NIRG-like cells in the ON	numerous	none	none	many	many
GFAP+ glia in the ONH	numerous	numerous	none	numerous	numerous

ONH – Optic Nerve Head, ON – optic nerve.

**Table 2:** Summary of immunolabeling of glial cells in the retina and optic nerve of different vertebrate species.

RETINA	Chicken	Mouse	Guinea Pig	Dog	Monkey
**GFAP**	Astrocytes	Astrocytes	No labeling	Astrocytes	Astrocytes
**S100β**	No labeling	Astrocytes	Müller glia	No labeling	Müller glia ∼half
**vimentin**	Müller glia & NIRG cells	Müller glia end-feet & astrocytes	Müller glia end-feet	Müller glia end-feet	Müller glia end-feet astrocytes
**Nestin/transitin**	NIRG cells	No labeling	Not done	Not done	NIRG cells
**Nkx2.2**	NIRG cells & Oligodendrocytes	No labeling	No labeling	No labeling	No labeling
**Sox2**	Müller glia Cholinergic amacrine cells & NIRG cells	Müller glia, Cholinergic amacrine cells & astrocytes	Müller glia Cholinergic amacrine cells	Müller glia, Astrocytes, Cholinergic amacrine cells & NIRG cells	Müller glia, Astrocytes, Cholinergic amacrine cells & NIRG cells
**Sox9**	Müller glia NIRG cells Oligodendrocytes	Müller glia & Astrocytes	Müller glia	Müller glia, Astrocytes & NIRG cells	Müller glia, Astrocytes & NIRG cells

OPTIC NERVE	Chicken	Mouse	Guinea Pig	Dog	Monkey
**GFAP**	Astrocytes	Peripapillary glia & Astrocytes	Astrocytes	Astrocytes	Astrocytes
**S100β**	No labeling	Peripapillary glia & Astrocytes	Peripapillary glia & optic nerve glia	No labeling	Peripapillary glia & optic nerve glia
**vimentin**	Astrocytes & Peripapillary glia,	Not done	Not done	Astrocytes	Astrocytes
**Nestin/transitin**	NIRG cells	No labeling	Not done	Not done	Glia in the ONH
**Nkx2.2**	Glia in the ONH & optic nerve	No labeling	Optic nerve glia	Optic nerve glia & ONH glia	Optic nerve glia
**Sox2**	Peripapillary glia & optic nerve glia	Peripapillary glia & optic nerve glia	Peripapillary glia & optic nerve glia	Peripapillary glia & optic nerve glia	Peripapillary glia & optic nerve glia
**Sox9**	Peripapillary glia & optic nerve glia	Peripapillary glia & optic nerve glia	Peripapillary glia & optic nerve glia	Peripapillary glia & optic nerve glia	Peripapillary glia & optic nerve glia

ONH – Optic Nerve Head.

### Glial cell in the mouse eye

**Figure 2:**
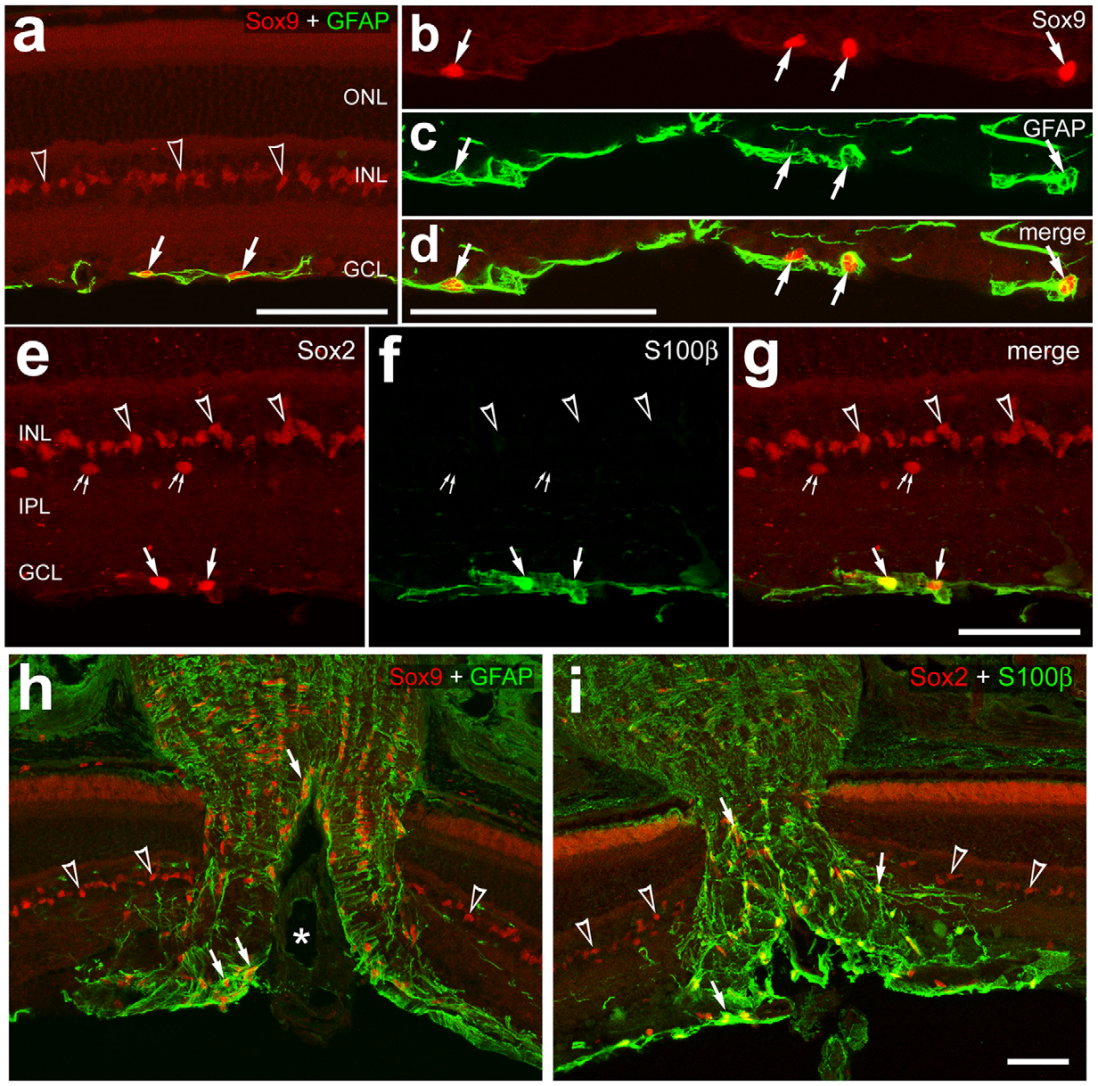
Glial cells in the mouse retina and optic nerve are immunoreactive for Sox2, Sox9, GFAP, and S100β. Sections through the retina and optic nerve head were labeled with antibodies to Sox9 (magenta; **a–d** and **h**), GFAP (green; **a–d** and **h**), Sox2 (magenta; **e–g** and **i**), and S100β (green; **e–g** and **i**). Hollow arrow-heads indicate the nuclei of Müller glia. Arrows indicate the nuclei of astrocytes. Small double-arrow-heads indicate the nuclei of cholinergic amacrine cells that are labeled for Sox2. Asterisks indicate blood vessels. The scale bar (50 µm) in panel **a** applies to **a** alone, the bar in **d** applies to **b–d**, the bar in **g** applies to **e–g**, and the bar in **i** applies to **i** and **h**. Abbreviations: ONL – outer nuclear layer, INL – inner nuclear layer, IPL – inner plexiform layer, GCL – ganglion cell layer.

The NIRG cells in the chick retina express the transcription factors Sox2, Sox9 and Nkx2.2. Thus, we began by assaying for Sox2, Sox9 and Nkx2.2 in the adult mouse retina to test whether NIRG cells are present in this tissue. Although we failed to detect Nkx2.2 in the mouse retina, we found widespread expression of Sox2 and Sox9; these factors were present in the nuclei of Müller glia (Figs. 2a and 2e), consistent with previous reports [12], [13]. In addition, Sox2 and Sox9 were present in the nuclei of S100β/GFAP-positive astrocytes in inner retinal layers (Figs. 2a-g). All (n = 187) of the Sox9-positive nuclei within the ganglion cell layer and NFL were co-labeled for GFAP. Similarly, all (n = 127) of the Sox2-positive nuclei within the GCL and NFL were labeled for S100β. In addition, we observed Sox2-immunoreactivity in the nuclei of cholinergic amacrine cells in the vitread INL and displaced to the GCL; these cells were co-labeled for Islet1 (data not shown). Co-labeling for Islet1 and Sox2 was used to distinguish displaced cholinergic amacrine cells in the GCL; Islet1 is expressed by cholinergic retinal amacrine cells in all birds and mammals [23], [24], [25]. Within the optic nerve and nerve head of the mouse eye, numerous astrocytes were labeled for Sox9 and GFAP or Sox2 and S100β (Figs. 2h and 2i). We did not detect Nkx2.2-positive cells in the ONH or optic nerve, within 1 mm of the retina. Thus, NIRG-like cells may not be present in the eyes of mice. See table 1 for a summary of markers expressed by glial cells in the mouse eye, and table 2 for a summary of the types of glial cells in the retina, optic nerve and nerve head.

### Glial cells in the guinea pig eye

**Figure 3:**
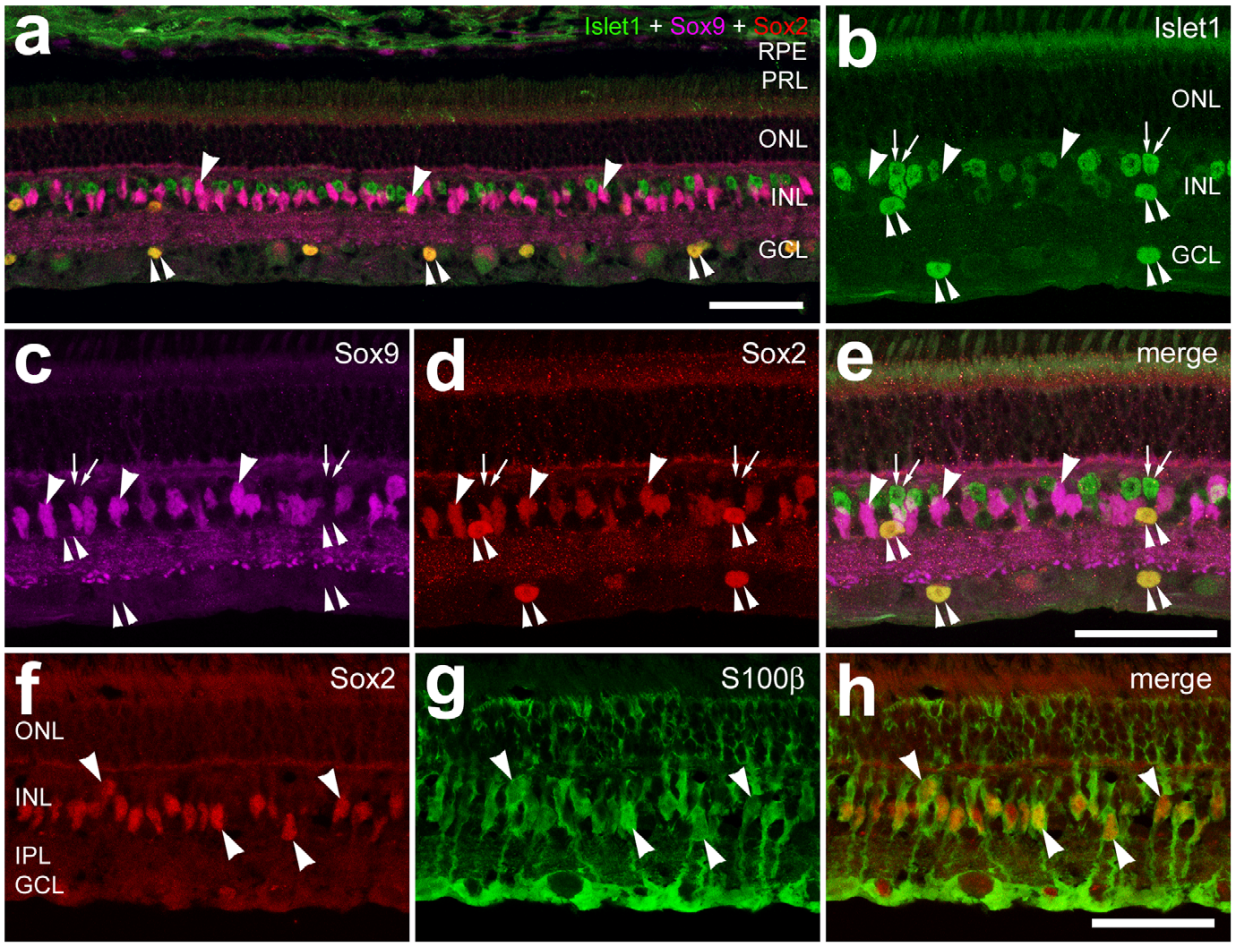
The guinea pig retina does not contain astrocytes or NIRG cells. Vertical sections of the retina were labeled with antibodies to Islet1 (green; **a**, **b** and **e**), Sox9 (magenta; **a**, **c** and **e**), Sox2 (red; **a**, **d–f** and **h**) and S100β (green; **g** and **h**). Arrow-heads indicate the nuclei of Müller glia, small double-arrows indicate the nuclei of bipolar cells that are labeled for Islet1 alone, and the small double-arrow-heads indicate the nuclei of cholinergic amacrine cells that are labeled for Islet1 and Sox2. The scale bar (50 µm) in panel **a** applies to **a** alone, the bar in **b** applies to **b–e**, and the bar in **h** applies to **g–h**. Abbreviations: RPE – retinal pigmented epithelium, PRL – photoreceptor layer, ONL – outer nuclear layer, INL – inner nuclear layer, IPL – inner plexiform layer, GCL – ganglion cell layer.

To assay for NIRG cells in the guinea pig retina we labeled tissues with antibodies to Sox2, Sox9 and Islet1; presumptive NIRG cells in the IPL or GCL should be positive for Sox2 and Sox9, but not Islet1. Immunoreactivity for Islet1 was detected in the nuclei of presumptive bipolar cells, ganglion cells and sparsely distributed cholinergic amacrine cells (Figs. 3a and 3b). Immunoreactivity for Sox9 was found only in the nuclei of Sox2^+^ Müller glia (Figs. 3c-e). In addition to the Mülller glia, antibodies to Sox2 labeled the nuclei of orthotopic and displaced cholinergic amacrine cells that were positive for Islet1 (Figs. 3a-e). To further probe for glial cells we labeled retinal sections for S100β. We found that all of the fusiform Sox2-positive nuclei in the middle of the INL were those of S100β-expressing Müller glia (Figs. 3g-h). There were no cells that were labeled for Sox2 and Sox9 in the IPL, GCL or NFL, suggesting that NIRG cells are not present in the guinea pig retina. GFAP-immunofluorescence was not detected in the guinea pig retina (data not shown), suggesting the absence of astrocytes within the neural retina. The absence of GFAP-expressing cells in the guinea pig retina is consistent with previous reports [14], [26], [27].

**Figure 4:**
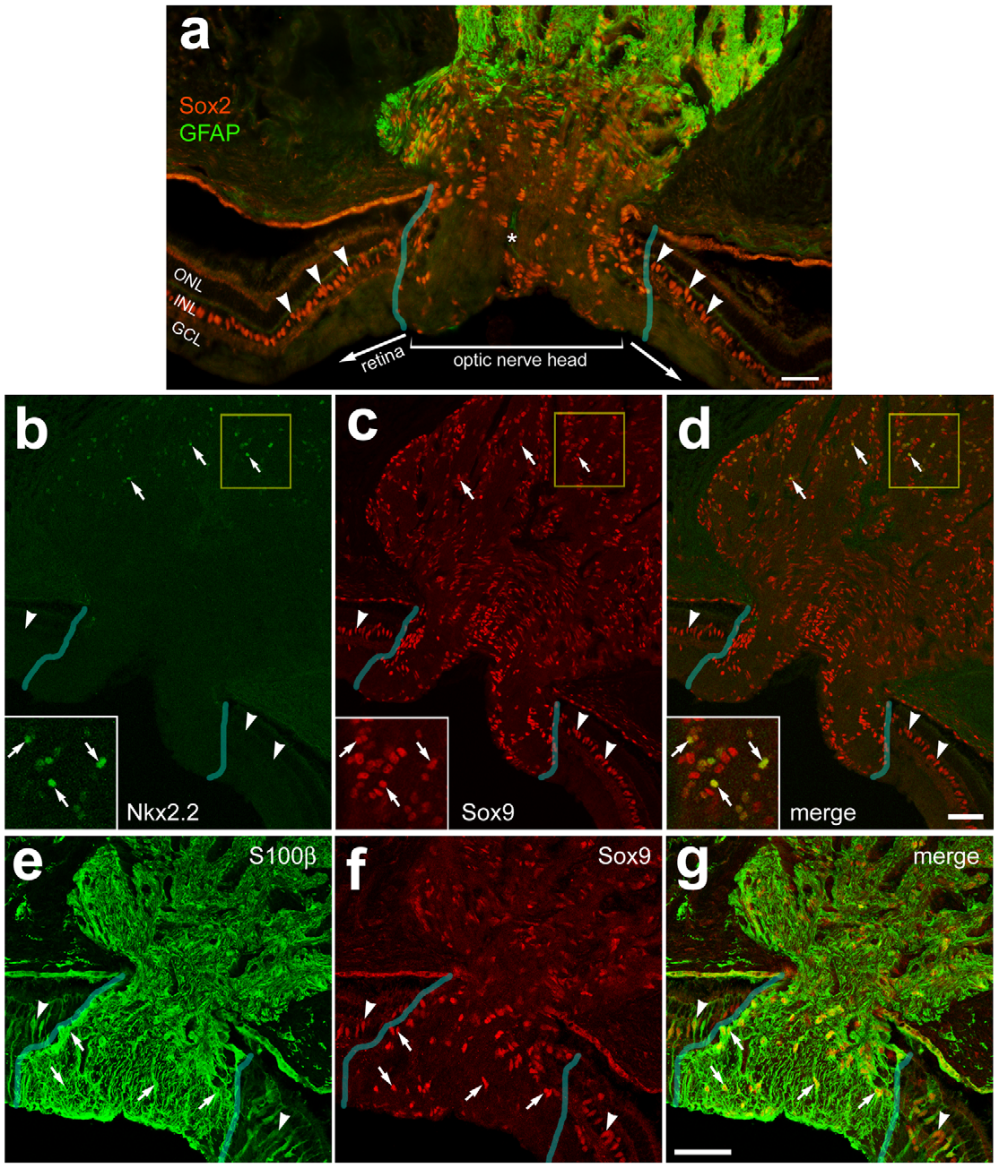
Glial cells in the guinea pig optic nerve and nerve head are immunoreactive for Sox2, GFAP, Nkx2.2, Sox9 and S100β. Longitudinal sections through the optic nerve and nerve head were labeled with antibodies to Sox2 (red in **a**), GFAP (green in **a**), Nkx2.2 (green in **b** and **d**), Sox9 (red in **c**,**d**,**f** and **g**) and S100β (green in **e** and **g**). Images were obtained by using wide-field epifluoresence microscopy (**a**) or confocal microscopy (**b–g**). Arrow-heads indicate the nuclei of Müller glia. The arrows in panels **b–d** indicate glial cells in the optic nerve that are immunoreactive for Nkx2.2 and Sox9. The arrows in panels **e–g** indicate glial cells in the optic nerve head that are immunoreactive for S100β and Sox9 The asterisks in panel **a** indicate a blood vessel. The regions indicated by yellow boxes in panels in **b–d** are enlarged 2-fold in the in-sets. The transparent blue lines indicate the boundaries between the optic nerve head and neural retina. The scale bar (50 µm) in panel **a** applies to **a** alone, the panel in **d** applies to **b–d**, and the bar in **g** applies to **e–g**. Abbreviations: ONL – outer nuclear layer, INL – inner nuclear layer, IPL – inner plexiform layer, GCL – ganglion cell layer.

We next probed for glial cells in the optic nerve and nerve head of the guinea pig eye. We found numerous cells that expressed Sox2 and Sox9 within the ONH; these cells did not express GFAP or Nkx2.2 (Figs. 4a-d). Interestingly, cells that expressed GFAP and/or Nkx2.2 were observed within the optic nerve; these cells were located approximately 300 µm posterior to the vitread surface of the ONH (Figs. 4a-d). In the optic nerve, all (n = 164) of the Nkx2.2-positive cells were immunoreactive for Sox9. The identity of the GFAP/Sox9/Nkx2.2-expressing cells in the optic nerve of the guinea pig remains uncertain, but these cells are not orthologous to the avian NIRG cells given the expression of GFAP. Within the nerve head and the optic nerve, all of the Sox9-positive cells were immunoreactive for S100β, consistent with the hypothesis that these were glial cells. See table 1 for a summary of markers expressed by glial cells in the guinea pig eye, and table 2 for a summary of the types of glial cells in the retina, optic nerve and nerve head.

### Glial cells in the dog eye

**Figure 5:**
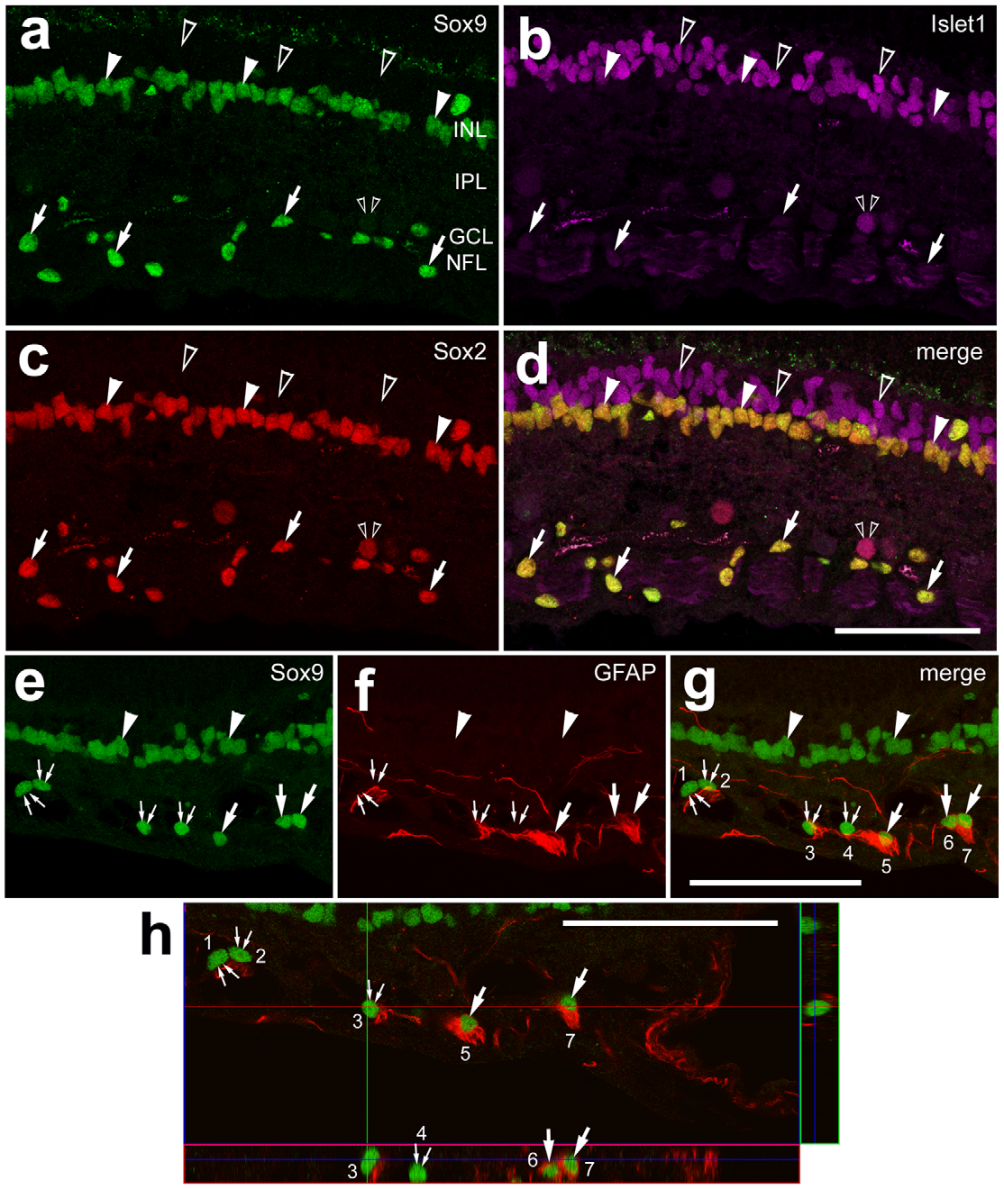
In the dog retina, astrocytes are immunoreactive Sox2, Sox9 and GFAP, whereas NIRG-like cells are immunoreactive Sox2 and Sox9 alone. Vertical sections of the retina were label with antibodies to Sox9 (green in **a**,**d**,**e**,**g** and **h**), Islet1 (magenta in b and d), Sox2 (red in c and d) and GFAP (red in **f–h**). Panel **h** includes orthogonal projections to demonstrate that some of the Sox9-positive nuclei in the NFL are not rimmed by GFAP-positive cytoplasm. Arrow-heads indicate the nuclei of Müller glia. Hollow arrow-heads indicate Islet1-positive nuclei of bipolar cells. Small hollow, double arrow-heads indicate the nucleus of cholinergic amacrine cells that is positive for Islet1 and Sox2. Small double-arrows indicate the nuclei of presumptive NIRG-like cells that are positive for Sox9 and Sox2, but negative for GFAP. Arrows indicate GFAP-positive astrocytes that are labeled for Sox9 and Sox2. The scale bar (50 µm) in panel **d** applies to **d** and **a–d**, the bar in **g** applies to **e–g**, and the bar in **h** applies to **h** alone. Abbreviations: INL – inner nuclear layer, IPL – inner plexiform layer, GCL – ganglion cell layer, NFL – nerve fiber layer.

The expression patterns of Islet1, Sox2 and Sox9 in dog retina and optic nerve were similar to patterns seen in chick, mouse and guinea pig eyes. For example, immunoreactivity for Islet1 was observed in the nuclei of bipolar cells in the distal INL and in presumptive cholinergic amacrine cells in the proximal INL and GCL that were positive for Sox2 (Figs. 5a-d). We observed immunoreactivity for Sox2 and Sox9 in the nuclei of Müller glia and in the nuclei of cells that were scattered across the GCL and NFL (Figs. 5a-d). Approximately half of the Sox9^+^ cells in the GCL and NFL were immunoreactive for GFAP (Figs. 5e-h), indicating that these cells were astrocytes. Levels of GFAP-immunoreactivity were not detectable in the Müller glia in normal, healthy dog retinas. More than half (52.5±7.7%; n = 225 cells) of the Sox9-positive cells in the GCL were negative for GFAP. The identity of the Sox9^+^/GFAP^−^ cells in the GCL and NFL of the dog retina remains uncertain. There was no labeling for Nkx2.2 in the dog retina (not shown). The Sox2^+^/Sox9^+^ cells in the dog retina were negative for PCNA (data not shown), indicating that these cells were post-mitotic.

**Figure 6:**
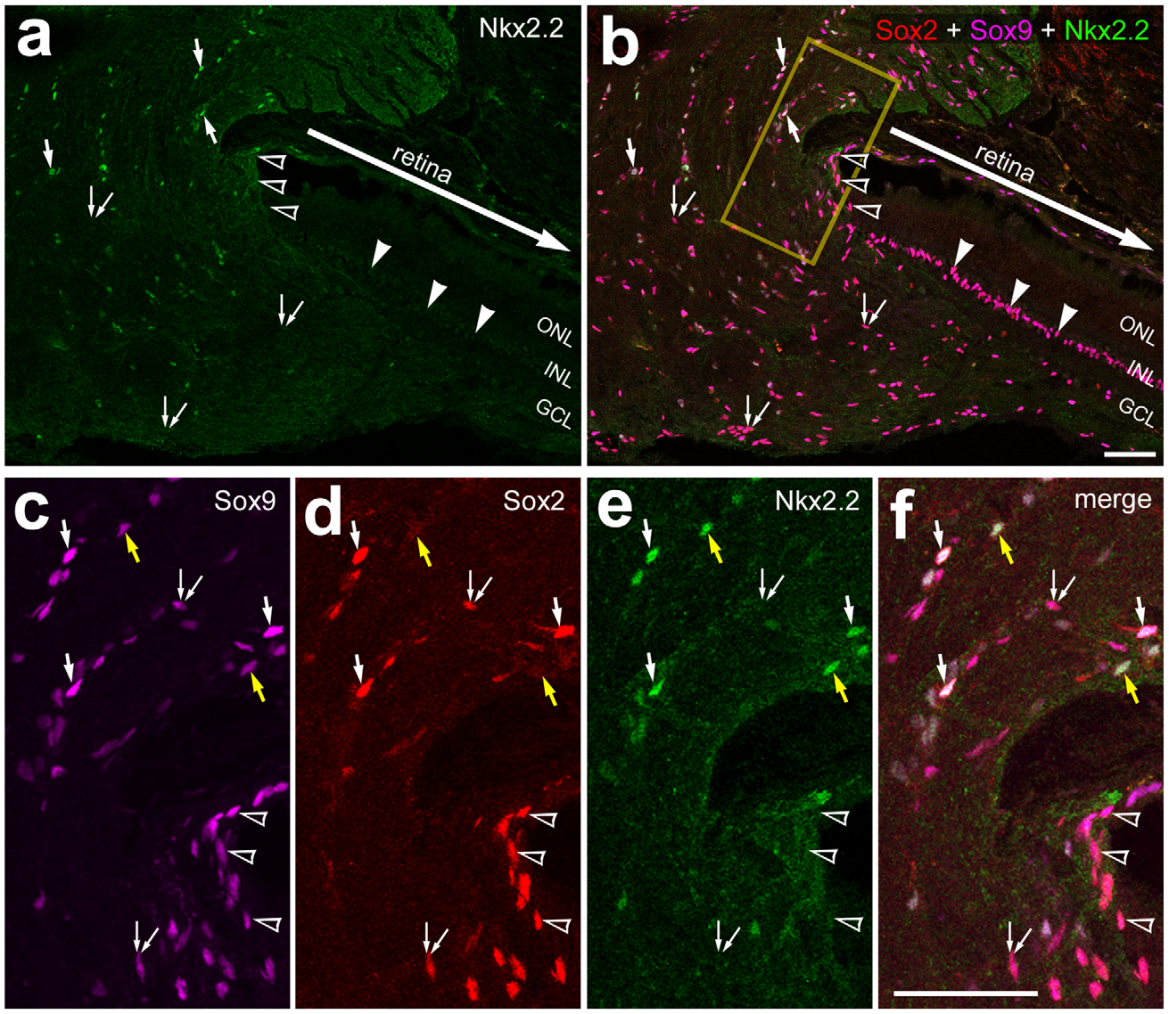
In the dog eye, glial cells in the optic nerve and nerve head express the transcription factors Nkx2.2, Sox2 and Sox9. Longitudinal sections through the optic nerve and nerve head were labeled with antibodies to Nkx2.2 (green), Sox2 (red) and Sox9 (magenta). The area indicated by the yellow box in panel **b** is enlarged 2.5-fold in panels **c–f**. White arrows indicate the nuclei of cells labeled for Nkx2.2, Sox 2 and Sox9. Yellow arrows indicate cells labeled for Nkx2.2 and Sox9, but not Sox2. Small double-arrows indicate cells labeled for Sox2 and Sox9, but not Nkx2.2. Solid arrow-heads indicate the nuclei of Müller glia labeled for Sox2 and Sox9. Hollow arrow-heads indicate peripapillary glia labeled for Sox2 and Sox9. The scale bar (50 µm) in panel **b** applies to **a** and **b**, and the bar in **f** applies to **c–f**. Abbreviations: ONL – outer nuclear layer, INL – inner nuclear layer, GCL – ganglion cell layer.

In the optic nerve and nerve head of dog eyes, we found numerous cells that were immunoreactive for Sox2 and Sox9. Nearly all (95.2±4.1%) of the Sox9-positive cells were immunoreactive for Sox2, and all of the Sox2-positive cells were immunoreactive for Sox9 (Fig. 6). Nearly half (44.3±8.5%) of the Sox9-positive cells were immunoreactive for Nkx2.2 (Figs. 6a-f). Many of Sox2/Nkx2.2-positive cells in the optic nerve and nerve head were negative for GFAP (not shown), suggesting that these cells may have been NIRG cells. The peripapillary glia were positive for Sox2 and Sox9, but negative for Nkx2.2 (Figs. 6c-f). See table 1 for a summary of markers expressed by glial cells in the dog eye, and table 2 for a summary of the types of glial cells in the retina, optic nerve and nerve head.

### Glial cells in the monkey eye

**Figure 7:**
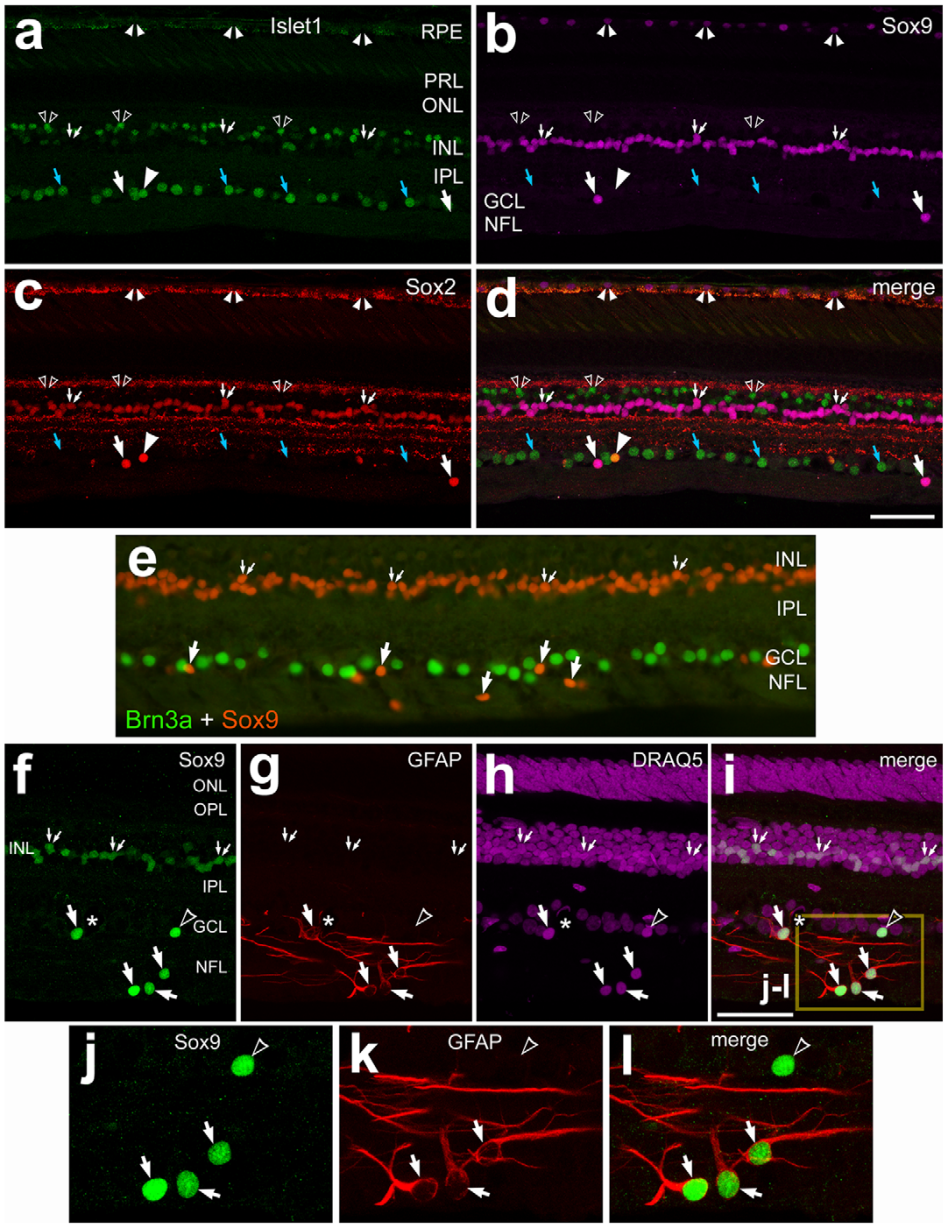
In the monkey eye, Sox2/Sox9-positive glial cells scattered across the GCL and NFL are GFAP^+^ astrocytes or GFAP^−^ NIRG-like cells. Vertical sections of the retina were labeled for Islet1 (green in **a** and **d**), Sox9 (magenta in **b** and **d**; red in e; green in **f–l**), Sox2 (red in **c** and **d**), Brn3a (green in **e**), GFAP (red in **g–l**). DRAQ5 (magenta) was used to stain nuclei (**h** and **i**). The area indicated by the yellow box in panel **i** is enlarged 2.5-fold in panels **j–l**. Small-double arrows indicate the nuclei of Müller glia. Arrows indicate the nuclei of glial cells in the GCL or NFL. Small double arrow-heads indicate Sox9-positive nuclei in the RPE (**a–d**). Small hollow, double arrow-heads indicate Islet1-positive nuclei of bipolar cells (**a–d**). Blue arrows indicate Islet1-positive nuclei of ganglion cells (**a–d**). In panels **f–l**., arrows indicate GFAP/Sox9-positive astrocytes and hollow arrow-heads indicate GFAP-negative/Sox9-positive NIRG-like cells. Asterisks indicate blood vessels. The scale bar (50 µm) in panel **d** applies to panels **a–d**, the bar in **e** applies to **e** alone, and the bar in i applies to **f–i**. Abbreviations: RPE- retinal pigmented epithelium, PRL – photoreceptor layer, ONL – outer nuclear layer, INL – inner nuclear layer, IPL – inner plexiform layer, GCL – ganglion cell layer, NFL – nerve fiber layer.

Patterns of expression for Islet1, Sox2 and Sox9 in monkey retina were similar to those observed in the eyes of other species that we examined. Islet1 was detected in the nuclei of bipolar cells, ganglion cells and cholinergic amacrine cells (Figs. 7a and 7d). Immunolabeling for Islet1, Sox2 and Sox9 revealed many cells scattered across the GCL and NFL that were immunoreactive for both Sox2 and Sox9, but negative for Islet1 (Figs. 7a-d). All (n = 271) of the Sox9-positive cells in the GCL or NFL were immunoreactive for Sox2 (Figs. 7a-d). The Sox2^+^/Sox9^−^ cells in the GCL were Islet1^+^, suggesting that these cells were displaced cholinergic amacrine cells (Figs. 7a-d). We tested whether any of the Sox9-positive cells in the GCL were some type of ganglion cell by combining labeling for Sox9 and Brn3a. Brn3a is known to be expressed by ∼98% of ganglion cells [28]. There was no overlap of labeling for Sox9 and Brn3a (Fig. 7a), suggesting that none of the Sox9-positive cells in the GCL were ganglion cells.

**Figure 8:**
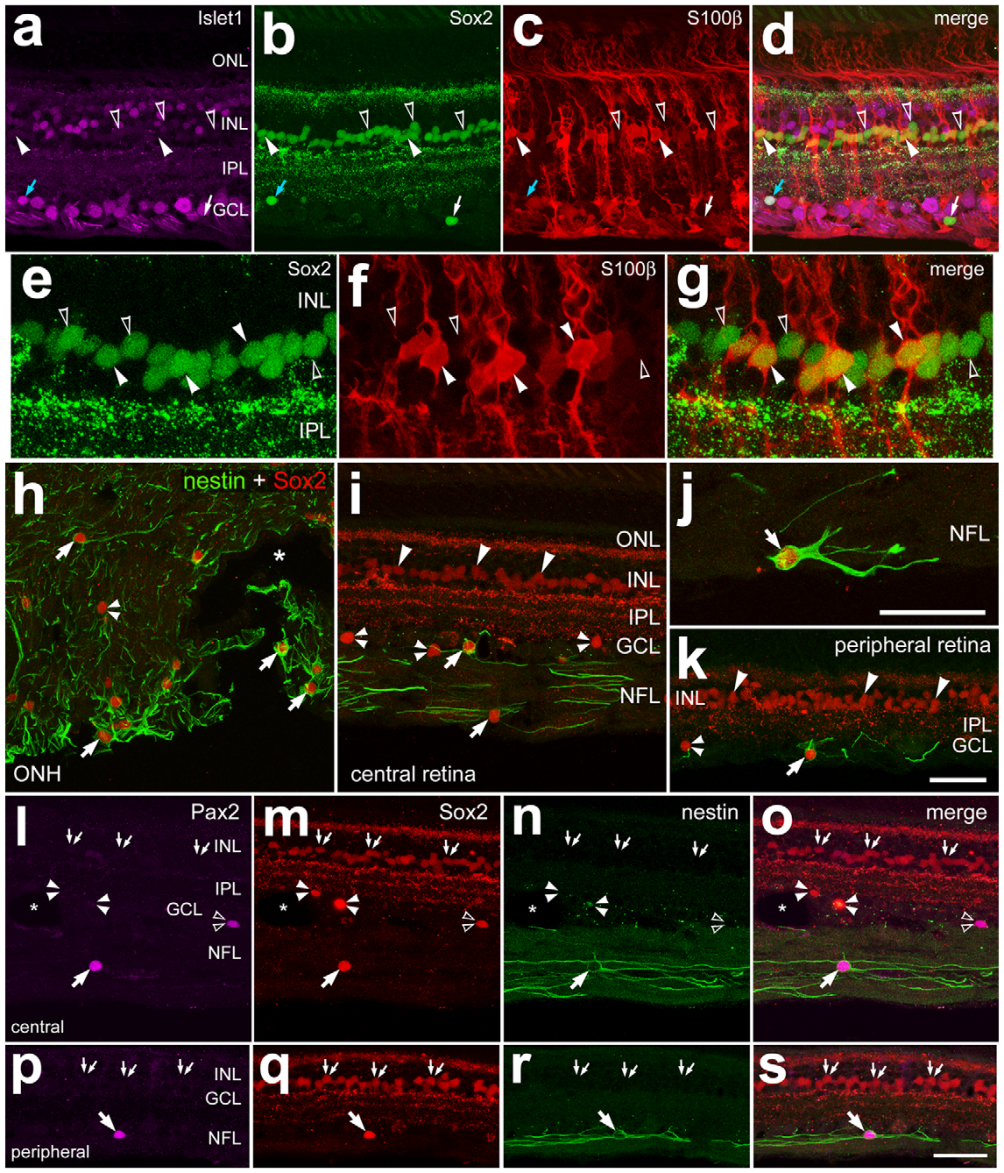
In the monkey eye, S100β is expressed by many Müller glia in the retina and glial cells in the optic nerve. Sections of the retina and optic nerve head were labeled with antibodies to Islet1 (**a** and **d**), Sox2 (green in **b**,**d**,**e** and **g**; red in **h–k**), S100β (red in **c**,**d**,**f** and **g**) and nestin (green in **h–k**). Arrow-heads indicate the nuclei of Müller glia labeled for Sox2 and/or S100β. Blue arrows indicate Sox2/Islet1-positive nuclei of cholinergic amacrine cells (**a–d**). Small double-arrow-heads indicate the nuclei of Sox2-positive glial cells that are nestin-negative (**h–o**). Arrows indicate presumptive NIRG-like cells that are labeled for Sox2, nestin and Pax2. Small hollow-double-arrows indicate nuclei labeled for Pax2 and Sox2, but not nestin (**l–o**). Asterisks indicate blood vessels. The scale bar (50 µm) in panel **d** applies to **a–d**, the bar in **h** applies to **e–g** and **h**, and the bar in **i** applies to **f**, **g** and **i**. Abbreviations: ONL – outer nuclear layer, INL – inner nuclear layer, IPL – inner plexiform layer, GCL – ganglion cell layer, NFL – nerve fiber layer, ONH – optic nerve head.

More than half (58.3±9.4%; n = 164 cells) of the Sox9-positive cells in the GCL were positive for GFAP (Fig. 7f-l), indicating that Sox9 is expressed by mature astrocytes. However, this finding also suggests that the Sox9+/GFAP- cells in the GCL and NFL are some type of non-astrocytic glial cell. To assess this possibility we probed for additional glial markers. Since the astrocytes in the mouse retina (Fig. 2) and dog retina (not shown) express S100β, we examined whether the Sox2^+^/Sox9^+^ cells in the GCL and NFL of the monkey retina were positive for S100β. Surprisingly, we found that the patterns of S100β expression in the monkey retina were not similar to those seen in the retinas of mice and dogs, but were similar to those seen in the retinas of guinea pigs. Nearly half (46.1±8.1%; n = 201 cells) of the Sox2-positive Müller glia were co-labeled for S100β (Figs. 8a-g). Interestingly, not all of the Müller glia were labeled for S100β, and S100β was not detected other types of glial cells in the primate retina.

Since the NIRG cells in the chick retina normally express transitin, the avian ortholog of mammalian nestin, we examined whether nestin was expressed in primate retina. Scattered across the NFL in central and peripheral regions of the retina, we detected many cells that were immunoreactive for Sox9 and nestin (Figs. 8g-i). The processes of nestin-positive cells tended to project parallel to the vitread surface of the retina (Figs. 8g and 8h). In addition, we observed numerous cells within the ONH that were immunoreactive for both nestin and Sox9 (Fig. 8f). A recent report has demonstrated that Pax2 is expressed by glial cells in the adult primate retina; nearly half of the Pax2-positive cells in the GCL or NFL are GFAP-expressing astrocytes [14]. We found that more than one-third (39.1±8.6%) of the Pax2^+^/Sox2^+^ cells in the GCL or NFL expressed nestin (Figs. 8l-s). The Sox2^+^/Pax2^−^ cells in the GCL were putative displaced cholinergic amacrine cells (Figs. 8l-s). The Sox2^+^/Sox9^+^ cells in the primate retina were negative for PCNA (data not shown), indicating that these cells were post-mitotic.

**Figure 9:**
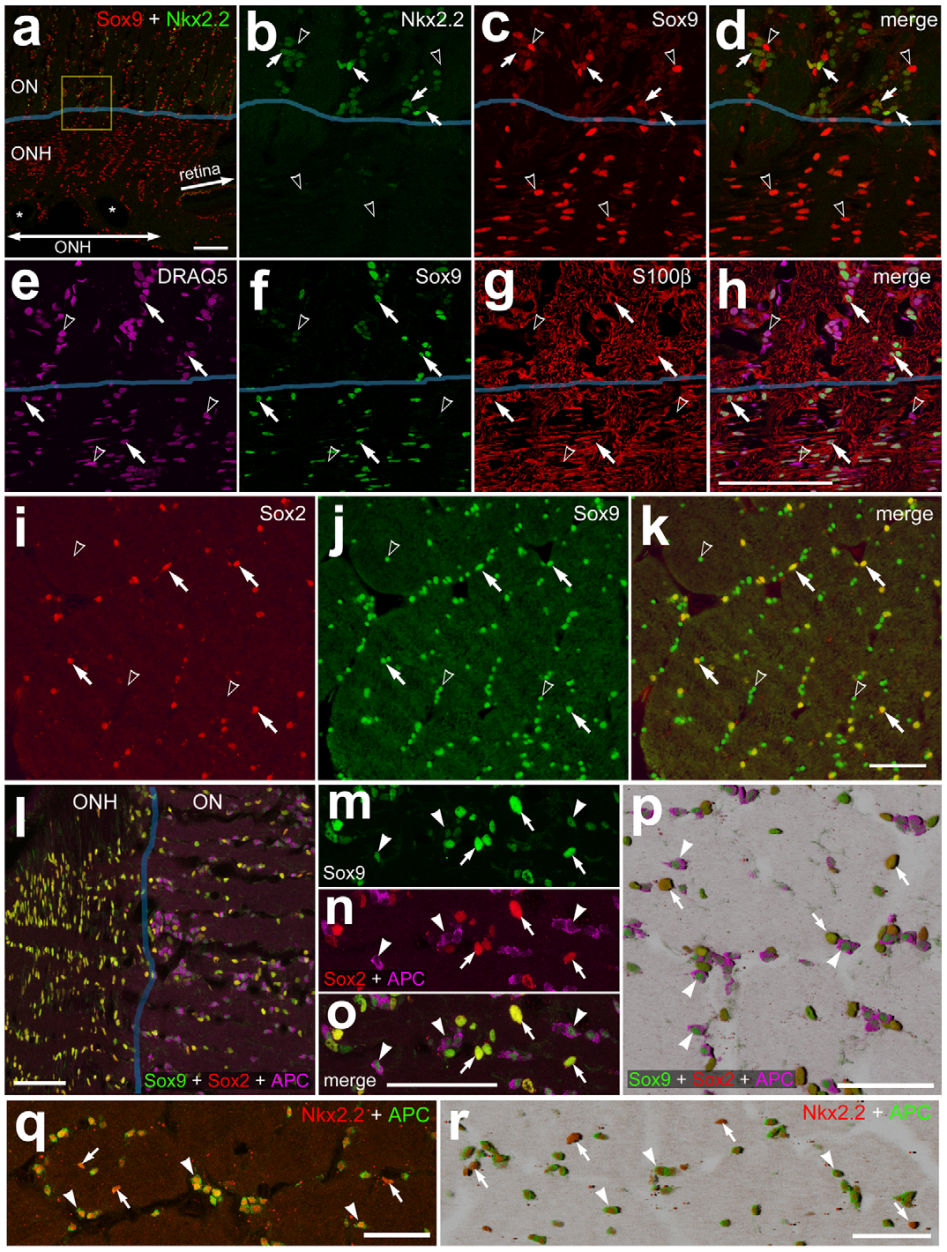
In the monkey eye, glial cells in the optic nerve and nerve head express Sox9, Nkx2.2, Sox2, S100β and APC. Longitudinal and transverse sections through the optic nerve and nerve head were labeled with antibodies to Sox9 (red in **a–d**; green in **e–k** and **l–p**), Nkx2.2 (green in **a–d**; red **q** and **r**), S100β (red in **g** and **h**), Sox2 (red in **i**, **k** and **l–p**) and APC (magenta **l–p**; green **q** and **r**). DRAQ5 (magenta) was used to stain nuclei (**e** and **h**). Images were obtained using confocal microscopy (**a–h** and **l–r**) or wide-field epifluoresence (**i–k**). The images in panels **p** and **r** were generated as 3D shadow-reconstructions of Z-stacks using Zeiss Zen software. The area indicated by the yellow box in panel **a** is enlarged 4-fold in panels **b–d**. Arrows indicate glial cells in the optic nerve that are labeled for Nkx2.2 and Sox9 (**a–d**), Sox9 and S100β (**e–h**), Sox9 and Sox2 (**i–k**), Sox2, Sox9 but not APC (**l–p**), or Nkx2.2 and APC (**q** and **r**). Hollow arrow-heads indicate the nuclei of glial cells labeled for Sox9 alone (**b–d** and **i–k**), or DRAQ5 alone (**e–h**). Arrowheads indicate oligodendrocytes labeled for Sox2, Sox9 and APC (**l–p**) or Nkx2.2 and APC (**q** and **r**). Asterisks indicate blood vessels. The scale bar (50 µm) in panel **a** applies to **a** alone, the bar in **h** applies to **e**–**h**, the bar in **k** applies to **i–k**, the bar **l** applies to **l** alone, the bar **p** applies to **m–p** alone, the bar in **q** applies to **o** alone, the bar in **r** applies to **r** alone.

Since the NIRG cells in the chick retina express Nkx2.2, we probed for Nkx2.2 in the glial cells of the monkey retina. We failed to detect Nkx2.2^+^ cells within the monkey retina (not shown). However, similar to the guinea pig eye, we detected numerous Nkx2.2^+^ cells in the optic nerve immediately posterior to the ONH (Figs. 9a-d). The Nkx2.2^+^ cells appeared approximately 250 µm posterior to the vitread surface of the ONH (Fig. 9a). Most of the Nkx2.2^+^ cells in the optic nerve were weakly immunoreactive for Sox9 (Figs. 9a-d). Most, if not all, of the Sox9^+^ cells in the optic nerve were immunoreactive for S100β (Figs. 9e-h), indicating that these cells are glial. Within the ONH, there was a complete overlap of Sox2 and Sox9 in the nuclei of cells (Fig. 9l). By comparison, within the optic nerve most of the Sox9^+^ cells were negative for Sox2, with only about one-third (39.9±6.8%) of these cells expressing Sox2 (Figs. 9m-p). This finding suggested that Sox9 is expressed by at least 2 distinct types of cells in the optic nerve. About two-third of the Sox9^+^ cells in the optic nerve were immunoreactive for APC, a marker that is known to be expressed by mature oligodendrocytes in the mammalian CNS [29]. The levels of Sox9-expression were relatively low in the APC^+^ oligodendrocytes (Figs. 9m-o). The APC^+^ oligodendrocytes did not express Sox2, were not seen within the ONH, but were found within the optic nerve immediately posterior to the ONH (Figs. 9l-p), similar to the distribution of Nkx2.2^+^ cells (Fig. 9a). Accordingly, we used sequential immunolabeling to determine whether APC^+^ oligodendrocytes in the optic nerve express Nkx2.2. We found that all of the APC^+^ oligodendrocytes were immunoreactive for Nkx2.2 (Figs. 9q and r). However, about one-eighth (13.3±6.1%) of the Nkx2.2^+^ cells in the optic nerve were not immunoreactive for APC, leaving the identity of Nkx2.2^+^/APC^−^ cells uncertain. See table 1 for a summary of markers expressed by glial cells in the monkey eye, and table 2 for a summary of the types of glial cells in the retina, optic nerve and nerve head.

## Discussion

We report here that glial cells in the retina and optic nerve of various vertebrate species have notable phenotypic differences, among distinct similarities. In all mammalian retinas, both astrocytes and Müller glia express Sox2 and Sox9. In addition, we have recently reported that astrocytes in the retinas and optic nerves of mice, guinea pigs, dogs and monkeys express the transcription factor Pax2 [14]. Without deviation, all mature, normal astrocytes express high levels of GFAP (reviewed by [30]), however the distribution and phenotype of astrocytes within the retina and optic nerve is variable between species. The retinas of chicks and guinea pigs contain few, if any, astrocytes [1], [8], [14]. By contrast, the retinas of mice, dogs and monkeys contain numerous astrocytes that are scattered across the NFL and GCL.

### Origins of NIRG cells

It is likely that the NIRG cells, similar to retinal astrocytes, migrate into the retina through the optic nerve. During development, retinal astrocytes migrate into the retina from the optic nerve [31], [32]. Interestingly, GFAP^+^ astrocytes in the guinea pig and Nkx2.2^+^ glial cells in guinea pigs and monkeys do not migrate beyond the optic nerve into the nerve head or neural retina. The migration of glial cells into the nerve head and retina is likely inhibited by mechanisms similar to those that halt the migration of oligodendrocytes from the optic nerve into the rodent and primate retina (see Fig. 9; reviewed by [33]). The glia within the optic nerve differentiate and migrate under the influence of the secreted factors such as Shh and Bone Morphogenetic Proteins (BMPs). Shh is provided by the growing axons of retinal ganglion cells to initiate the formation glia in the optic chiasm [34], [35], [36]. BMPs may be provided by the neural retina to suppress the migration of oligodendrocytes into the mammalian retina and/or ONH [37].

We identified NIRG cells in the optic nerve of the chicken, consistent with the hypothesis that these cells migrate into the retina from the optic nerve. A recent study from Rompani and Cepko [38] describes the developmental origins of the different types of glial cells in the chick eye. The authors describe an optic nerve-derived glial progenitor that gives rise to oligodendrocytes, astrocytes and a novel cell type that they termed “diacytes”. The authors describe presumptive astrocytes in the IPL and diacytes in the GCL based on developmental origins and morphology; the morphology of the diacytes and presumptive astrocytes are reminiscent of astro-glial cells. The diacytes and presumptive astrocytes express Olig2 (similar to oligodendrocytes), whereas these cells do not express other well-known glial markers including GFAP, myelin proteolipid protein, myelin/oligodendrocyte-specific protein or myelin-associated glycoprotein [38]. We propose that the astrocytes and diacytes described by Rompani and Cepko are the NIRG cells that we have described [1]. We determined that the NIRG cells/ astrocytes/diacytes were negative for well-established glial markers including GFAP, glutamine synthetase, 2M6 (Top_AP_), and transferrin-binding protein, whereas the NIRG cells were positive for Sox2, Sox9, Nkx2.2, vimentin and transitin. We propose that the astrocytes/diacytes/NIRG cells in the chicken retina are not a type of astrocyte because these cells are negative for both GFAP and Pax2, and these cells do not significantly up-regulate GFAP in response to retinal damage [1], [14], [38]. In all species examined, retinal astrocytes express GFAP and Pax2, and GFAP expression is dramatically increased in damaged tissues [14], [39]. Furthermore, we find conventional astrocytes in the chick optic nerve (but not retina) that express GFAP and Pax2, but not Nkx2.2 (current study and [14]), unlike the NIRG cells within the retina which are negative for GFAP and Pax2, and positive for Nkx2.2 [1]. These findings suggest that conventional optic nerve astrocytes fail to migrate beyond the optic nerve head into the chick retina. Taken together, we believe that the diacytes/astrocytes described by Rompani and Cepko are, are not conventional astrocytes. It is possible the NIRG cells in the chick retina represent two separate types of glial cell based on differences in morphology and laminar distribution within the retina, as described by Rompani and Cepko. A thorough functional analysis of these unusual glial cells in the chick retina is required to better define the phenotype of these cells.

### NIRG cells in the chick versus NIRG-like cells in the dog and monkey

The identity of the GFAP^−^/Sox2^+^/Sox9^+^ cells in the GCL and NFL in the retinas of primates and dogs remains somewhat uncertain, but is consistent with the hypothesis that these are NIRG-like cells. It is remotely possible that the GFAP^−^/Sox9^+^/Sox2^+^/nestin^+^/Pax2^+^ cells are displaced Müller glia or perhaps quiescent glial progenitors. Many studies have demonstrated that mature Müller glia express Sox2 and Sox9, similar to proliferating retinal progenitors [1], [9], [10], [12], [13]. Müller glia are known to express nestin in response to neuronal damage [40], [41], [42]. However, the nestin^+^ cells in the normal monkey retina likely were not reactive Müller glia given that observations were made in normal, healthy retinas, and the NIRG-like cells were Pax2-positive whereas the Müller glia were Pax2-negative. It is unlikely that the GFAP^−^/Sox2^+^/Sox9^+^/Pax2^+^/nestin^+^ cells are quiescent progenitors since there have been no compelling reports of on-going gliogenesis or neurogenesis in the dog or primate retina. Consistent with this notion, we did not detect PCNA expression in the glial cells in the retinas of mature dogs and monkeys. Nevertheless, we cannot exclude the possibility that some of these GFAP^−^/Sox2^+^/Sox9^+^/Pax2^+^/nestin^+^ cells are glial progenitors that are quiescent in normal, healthy retinas and become gliogenic in response to neuronal damage. However, it is worth noting that the expression of many “progenitor cell” transcription factors, such as Pax6, Chx10, Six3 and Rx, is maintained in different sets of mature retinal neurons (reviewed by [43], [44]). Thus, it is not surprising that “progenitor cell” transcription factors such as Sox2 and Sox9 are expressed by mature glial cells, such as astrocytes, NIRG-like cells and Müller glia.

Our findings indicate that the phenotype of NIRG cells in the chick is subtly different from that of NIRG-like cells in the dog and primate. Unlike the NIRG cells in the chicken retina, we find NIRG-like cells in the primate retina expressed Pax2, but not Nkx2.2. We have also reported that GFAP^−^/Sox2^+^/Pax2^+^ cells are scattered across inner layers of the dog retina [14]. In considering the above-listed findings, we propose that the primate retina contains NIRG-like cells that express Sox2, Sox9, Pax2 and nestin, but are negative for Nkx2.2, GFAP and S100β. Although many of the Sox2^+^/Sox9^+^ cells in the inner layers of the monkey retinas express nestin, labeling for nestin in the dog retina was not possible because of a paucity of antibodies with demonstrated specificity in dog tissues. Furthermore, in the chick retina the NIRG cells are scattered across the IPL, GCL and NFL [1], [14]. In the primate retina the NIRG-like cells are scattered across the GCL and NFL, but are never seen in the IPL (current study). In the chick retina, most of the NIRG cells are found in the sclerad half of the IPL, although many of these cells are also found scattered across the GCL, NFL and vitread half of the IPL, with transitin^+^ processes that tend to project horizontally within the retina [1]. By comparison, primate NIRG-like cells were found only in the GCL and NFL and extended nestin^+^ processes horizontally.

Based on our findings in the macaque retina, we propose that the human retina likely contains NIRG cells, given that the macaque retina is considered to be nearly identical to the human retina (reviewed by [45], [46], [47]). In the chick retina, the NIRG cells are thought to provide support to neurons and synapses in the IPL [1]. In the chick, when the NIRG cells are stimulated by insulin or IGF1 retinal neurons and the Müller glia are rendered more susceptible to excitotoxic damage [1]. Taken together, these findings suggest that NIRG cells in the human retina may contribute to the pathogenesis of diabetic retinopathy.

### Heterogeneity of glial cells and phenotypes between species

Our findings indicate that there is significant heterogeneity in the glial types and phenotypes of these glia in the retinas of different mammals. The variability of glial phenotypes is, in part, demonstrated by the patterns of expression of S100β in different glial cell types in the retinas of different mammals. For example, the retinal astrocytes in dogs and mice express S100β, whereas these cells do not express S100β in the monkey retina. Although the astrocytes in the monkey retina are negative for S100β, about half of the Müller glia are positive for S100β. By comparison, S100β is expressed by all of the Müller glia in the guinea pig retina, whereas S100β was not detected in the Müller glia of dogs or mice. S100β is a calcium-binding protein that is known to be expressed by astrocytes in rodents (reviewed by [48]). S100β has been shown to regulate intracellular calcium levels and promote the proliferation of astrocytes [49], [50]. Taken together, these findings suggest that the sub-set of Müller glia in the monkey retina expressing S100β may be predisposed to proliferate or may have an elevated requirement for calcium homeostasis.

There was a variable distribution of Nkx2.2-expressing cells in the eyes of different vertebrates. Unlike the NIRG cells in the chick retina, the NIRG-like cells in dog and primate retinas did not express Nkx2.2. In the monkey eye, similar to the guinea pig, Nkx2.2^+^ cells were detected in the optic nerve immediately posterior to the ONH. By comparison, the Nkx2.2^+^ cells in the dog eye were found in both the optic nerve and ONH, whereas Nkx2.2^+^ cells were not detected in the mouse eye. In the optic nerves of chicks, guinea pigs, dogs and monkeys, the Nkx2.2^+^ cells co-expressed Sox9. In the primate eyes, most of the Nkx2.2^+^ cells in the optic nerve were APC^+^ oligodendrocytes. In addition to APC^+^ oligodendrocytes, Nkx2.2 was also expressed by a population of APC-negative cells. The identity of the Nkx2.2^+^/APC^−^ cells remains uncertain. By comparison, the TFBP^+^ oligodendrocytes in the chick optic nerve were negative for Nkx2.2.

### Conclusions

We conclude that there is significant heterogeneity between the types of glial cells that are present in the retinas and optic nerves of warm-blooded vertebrates. Further, we find that the phenotypes of different, distinct types of glia vary with respect to the expression of Nkx2.2 and S100β. We further propose that NIRG-like cells are absent from the eyes of mice and guinea pigs, but may be present in significant numbers in the retinas of dogs and monkeys. In the primate retina, we identified significant numbers of prospective NIRG cells that expressed Sox2, Sox9, Pax2 and nestin, but were distinguished from astrocytes because of a stark absence of GFAP expression.

## Materials and Methods

### Animals

The use of animals in these experiments was in accordance with the guidelines established by the National Institutes of Health and the Weatherall report, “The use of non-human primates in research”. This study was approved by the Ohio State University IACUC (protocol 2009A0139). Newly hatched leghorn chickens (*Gallus gallus domesticus*) were obtained from the Department of Animal Sciences at the Ohio State University and kept on a cycle of 12 hours light, 12 hours dark (lights on at 7:00 am). Chicks were housed in a stainless steel brooder at about 25°C and received water and Purina^tm^ chick starter *ad libitum*.

In the current study, we used the eyes of six mice (*Mus musculata*; 4 months of age or older), four guinea pigs (*Cavia porcellus*; 4 months of age or older) and four dogs (*Canis familiaris;* between 2 and 6 years of age). The eyes were obtained post-mortem and were kindly provided by colleagues; mice from Dr. Karl Obrietan (Department of Neuroscience, The Ohio State University), guinea pigs from Dr. Jackie Wood (Department of Physiology and Cell Biology, Ohio State University), dogs from Dr. Simon Petersen-Jones (Veterinary Sciences, Michigan State University) and monkeys from Dr. John Buford (Department of Physiology and Cell Biology, The Ohio State University).

### Fixation, sectioning and immunocytochemistry

Tissues were fixed, sectioned and immunolabeled as described previously [40], [51], [52]. Sequential immunolabeling for primary antibodies raised in the same species was performed as described elsewhere [40], [53]. In short, double-labeling using two mouse monoclonal antibodies was performed over consecutive days, with the second primary antibody applied after the first secondary antibody. The first secondary antibody was expected to recognize only the first primary antibody, and the second secondary was expected to recognize both primary antibodies. None of the observed labeling appeared to be due to secondary antibody or fluorophore because sections labeled with secondary antibodies alone were devoid of fluorescence.

Working dilutions and sources of antibodies used in this study included the following: **(1)** mouse anti-Nkx2.2 was used at 1∶10 to 1∶50 (74.5A5; Developmental Studies Hybridoma Bank – DSHB; University of Iowa). The antiserum was raised to recombinant, full-length chick Nkx2.2 fused to GST [54]. The specificity of the Nkx2.2 antibody has been confirmed by an absence of labeling in Nkx2.2-/- mice [1], [55]. **(2)** goat anti-Sox2 was used at 1∶1000 (Y-17; Santa Cruz Biotechnology). The antibody was raised to the recombinant C-terminus of human Sox2 and recognizes a single 34 kDa band in Western blot analysis of lysate from mouse embryonic stem cells (manufacturer). The Sox2 antibody is known to recognize amino acids 277–293 of human Sox2, as determined by preabsorption controls and mass spectrometry analysis of blocking peptide [13]. **(3)** rabbit anti-TFBP (transferrin binding protein) was used at 1∶2000 (αOV-TfBP; Dr. J.J. Lucas, SUNY Upstate Medical University). The antibody was raised to chick oviduct TFBP and the specificity was confirmed by affinity chromatography and Western blot analysis which revealed a single band at 91 kDa [56]. **(4)** rabbit anti-Sox9 was used at 1∶2000 (AB5535; Millipore-Chemicon). The Sox9-antibody was raised to a synthetic peptide (VPSIPQTHSPQHWEQPVYTQLTRP) from human Sox9. The antibody detects a single band at ∼65 kDa by Western blot analysis (Manufacturer's technical information), and conditional knock-out of *Sox9* in the retina abrogates immunolabeling [13]. **(5)** mouse anti-glial fibrillary acidic protein (GFAP) was used at 1∶1000 (G-3893; Sigma-Aldrich). The antibody was raised to purified GFAP from porcine spinal cord and recognizes a single 52-kDa band in Western blot analysis (manufacturer). **(6)** rabbit S100β was used at 1∶100 (37A; Swant Immunochemicals). The antibody was raised to S100β that was purified from bovine brain. The specificity of the S100β antibodies has been confirmed by Western blots, ELISA, radioimmunoassay, and immunohistochemistry [57]. **(7)** mouse anti-Islet1 was used at 1∶50 (40.2D6; DSHB; University of Iowa). The Islet1 was raised to the C-terminus (amino acids 247–349) of rat Islet1. The antibody to Islet1 is known to recognize both Islet1 and Islet2 [58]. **(8)** mouse anti-nestin was used at 1∶100 (MAB5326, clone 10C2; Millipore-Chemicon). The antibody was raised to human nestin amino acids 1464–1614 fused to glutathione S-transferase [59]. The specificity of this antibody has been confirmed by Western blot analysis and revealed a single band at ∼220 kDa from protein extracts of human embryonic neural tissue [59], [60]. **(9)** mouse anti-vimentin was used at 1∶50 (40E-C; DSHB). This antibody was raised to homogenized adult canary brain and the specificity has been confirmed, with the detection of a single band at ∼50 kDa, by using Western blot analysis [61]. **(10)** rabbit anti-Pax2 was used at 1∶250 (PRB-276; Covance). The antibody was raised to amino acids (188–385) of human Pax2 and recognizes both Pax2a and Pax2b isoforms (manufacturer). The specificity of the Pax2 antibody was assessed by Western blot analysis, detecting 2 bands at 51 and 44 kDa, and by comparison of patterns of immunofluorescence to those seen with *in situ* hybridization [14]. **(11)** mouse anti-APC (*Adenomatous polyposis coli*) was used at 1∶500 (ab16794; Abcam). The monoclonal antibody was raised to recombinant human APC, amino acids 1–226. The specificity of the APC antibody has been assessed by Western blot analysis which demonstrated a single band at 300 kDa from rat brains [62]. **(12)** mouse anti-PCNA was used at 1∶1000 (clone PC10; Dako).

Secondary antibodies included donkey-anti-goat-Alexa488/568, goat-anti-rabbit-Alexa488/568/647, goat-anti-mouse-Alexa488/568/647, goat anti-rat-Alexa488 and goat-anti-mouse-IgM-Alexa568 (Invitrogen) diluted to 1∶1000 in PBS plus 0.2% Triton X-100.

### Photography, measurements, cell counts, and statistical analyses

Wide-field photomicrographs were obtained by using a Leica DM5000B microscope and Leica DC500 digital camera. Confocal images were obtained by using a Zeiss LSM510 at the Hunt-Curtis Imaging Facility in the Department of Neuroscience. Images were optimized for color, brightness and contrast, multiple-channel images overlaid, and figures constructed by using Adobe Photoshop™6.0. Cell counts were made from at least 5 different animals, and means and standard deviations calculated on data sets. To avoid the possibility of region-specific differences within the retina, cell counts were consistently made from the same region of retina for each data set.
